# Sequential Modulation of the Equine Fecal Microbiota and Fibrolytic Capacity Following Two Consecutive Abrupt Dietary Changes and Bacterial Supplementation

**DOI:** 10.3390/ani11051278

**Published:** 2021-04-29

**Authors:** Axelle Collinet, Pauline Grimm, Samy Julliand, Véronique Julliand

**Affiliations:** 1 Lab To Field, 21000 Dijon, France; axelle.collinet@lab-to-field.com (A.C.); pauline.grimm@lab-to-field.com (P.G.); samy.julliand@lab-to-field.com (S.J.); 2 Unité Mixte de Recherche Procédés Alimentaires et Microbiologiques (UMR PAM) A 02.102, AgroSup Dijon, University Bourgogne Franche-Comté, 21000 Dijon, France

**Keywords:** horse, hindgut ecosystem, dysbiosis, fiber degradation, lactic acid bacteria, lab probiotic, high starch diet

## Abstract

**Simple Summary:**

The equine hindgut is colonized by microorganisms, some of which are involved in fiber digestion and are crucial for the horse’s nutrition and health. These key microorganisms are very sensitive to dietary changes, which have been identified as a risk factor for colics. This study assessed the stressful effect of two consecutive abrupt dietary changes on the diversity, the composition, and the activity of fecal microorganisms focusing on fibrolytic bacteria. Twelve horses were subjected to an abrupt change from forage to a concentrate-rich diet, followed by a second change from a concentrate to forage-rich diet 5 days later. Half of the horses were given a supplement of living bacteria as a probiotic. Two days after the sudden change from forage to concentrate diet, the proportions and types of microorganisms were altered drastically, as was their capacity to degrade fibers. After this dietary stress, it took 3–4 weeks of a high-fiber diet to recover the basal state. Supplementation with probiotics promoted an earlier recovery of fibrolytic bacteria after the dietary stress. Abrupt dietary changes should be limited in horse management to protect the hindgut microorganisms and their capacity to use forage fibers, and consequently to limit the development of colic.

**Abstract:**

In horses, abrupt changes from high-fiber (HF) to high-starch (HS) diets can affect the cecal and colonic microbiota. This study investigated modifications and recovery of fecal microbiota after two consecutive abrupt dietary changes. Twelve horses fed HF for 2 weeks were changed to HS for 5 days then returned to HF for 7 weeks. Six received lactic acid bacteria supplementation. Bacterial population diversity, structure, and activity, especially fibrolysis, were assessed to obtain an overview of alteration in hindgut microbiota. Two days after the abrupt change from HF to HS, the findings in feces were consistent with those previously reported in the cecum and colon, with a decrease in fibrolytic activity and an increase in amylolytic activity. Fecal parameters stabilized at their basal level 3–4 weeks after the return to HF. A bloom of cellulolytic bacteria and lower pH were observed after 1.5 weeks, suggesting a higher level of fiber degradation. In supplemented horses the relative abundance of potentially fibrolytic genera was enhanced 2 days after HS and 2 days to 2–3 weeks after the return to HF. Fecal analysis could be a promising technique for monitoring hindgut microbial variations accompanying dietary changes.

## 1. Introduction

Colic has a high prevalence in the equine population [1] and is a leading cause of death in adult horses. Environmental changes such as recent changes of diet represent a major risk factor for the occurrence of colic [2]. Abrupt modifications of diet are commonly associated with changes in horse management practices such as moving stables for competition, reproduction, or clinical purposes. Julliand and Grimm reviewed evidence that the hindgut microbiota is quickly affected by abrupt dietary changes, especially by the sudden incorporation of a starch-rich concentrate within a high-fiber diet (HF) [3].

In 1978, a pioneer study demonstrated that feeding horses a sudden overload of starch and wood flour gruel induced increases in the cecal concentrations of *Lactobacilli* and *Streptococci* that were associated with a subsequent acidification [4]. Ten years later, another study reported an increase in the cecal concentrations of bacterial starch-utilizers within 24 h after an abrupt change from 100% chopped alfalfa hay to 100% concentrate [5]. By contrast, the proportions of xylanolytic and pectinolytic bacteria decreased markedly in the cecal content over the first several days [5]. Two studies conducted on larger numbers of individuals confirmed the increase in starch-utilizing bacteria in the cecum 24 h after a 100% forage diet was suddenly replaced by 30–50% concentrate diets [6,7]. Similar modifications were reported in the right ventral colon (RVC), associated with a consequent increase in lactic acid concentrations and concentrations of lactic acid-utilizing bacteria [7]. The fibrolytic activity was suggested to be affected because the number of bacteria able to use cellulose decreased in the cecum and colon 29 h after the diet change [6]. A study using high-throughput sequencing reported a decrease in the relative abundance of Fibrobacteraceae, a family containing key cellulolytic bacteria [8], in the cecum following an abrupt change from 100% hay to 0.6% or 1.2% of body weight (BW) of concentrate [9]. This study suggested an increase in the relative abundance of families able to degrade nonfiber carbohydrates (NFC) and to use lactic acid (Lactobacillaceae and Veillonellaceae, respectively) in association with a decrease in bacterial richness and diversity in the cecum in the first 12 h after the concentrate introduction. Three studies reported an alteration in bacterial activity concomitant with bacterial modifications with a shift in the proportions or concentrations of volatile fatty acids (VFAs) in the first 24 h after the abrupt dietary change [6,7,9]. Despite different experimental designs, diets and laboratory methods of analysis, the reported data indicate that an abrupt introduction of concentrate to a high-forage diet might generate alteration in bacterial richness, diversity, population structure, and activity in both the cecum and RVC ecosystems. The effects on the equine hindgut microbiota of a reverse change from high-starch (HS) to HF were evaluated by Goodson et al. [5] for 3 weeks after 6 weeks of concentrate feeding. Whether the bacterial population structure and the functions performed by the hindgut bacterial ecosystem return to the basal state after such a stress, and the kinetics of any changes, remain largely unknown.

Several strains of lactic acid bacteria (LAB) are regarded as probiotics and some may confer health benefits in adult horses. The supplementation with an LAB mixture increased the cellulolytic activity in the feces of mature horses [10] and enhanced cell-wall digestibility in the feces of endurance horses fed high amounts of forage [11]. Supplementation with another LAB mixture also contributed to the maintenance of fibrolytic activity and normal forage utilization in colonic contents even when horses were fed a HS diet [12]. *Lactobacillus acidophilus* alone or a mixture of other LAB mixture substantially reduced the risk of acidosis in horses whose dietary starch content was abruptly increased [13]. The manipulation of the hindgut microbiota with LAB could help minimizing feed-related changes in the microbiota and preventing some diseases.

We identified some limitations in the data of previous studies assessing the impact of abrupt dietary changes: (1) to our knowledge, microbial modifications after a sudden dietary change from forage to concentrate-rich diet have never been explored in feces; (2) only one study investigated the behavior of the hindgut ecosystem when a pony returns to its initial diet after the abrupt incorporation of concentrates; (3) data for cellulolytic bacteria are limited despite their key role in the horse’s nutrition and health; and (4) only one study used supplementation with LAB during an abrupt and massive incorporation of starch in the diet of horses.

In this in vivo study, 12 healthy adult horses were subjected to consecutive sudden changes from HF to HS diet and vice versa, to mimic the abrupt dietary modifications that can be encountered by horses. First, we aimed to evaluate modifications in feces, the most accessible material for studying the hindgut microbiota, and to compare with previously reported alterations in the proximal hindgut. Second, we monitored the modifications over 7 weeks after the sudden return to the initial HF diet to observe the recovery of the microbiota. Third, we evaluated the impacts of supplementation with a combination of three LABs as probiotics: *Lactobacillus acidophilus*, *Ligilactobacillus salivarius* (formerly *Lactobacillus salivarius* [14]), and *Bifidobacterium lactis*, during and after the two dietary stresses, for stabilizing the microbiota and supporting its recovery. We used a multidisciplinary approach to give a comprehensive picture of the microbiota modifications in terms of bacterial diversity, richness, population structure, composition, and activity. We focused on cellulolytic bacteria and their end-products to investigate in depth the impact of diet change on fibrolysis, which represents a key component of equine digestion and health.

## 2. Materials and Methods

All experimental procedures were evaluated and approved by the institutional ethics committee (Comité d’Ethique de l’Expérimentation Animale Grand Campus Dijon). The project was authorized by the French Ministry of Agriculture (registration number: APAFIS#18307-2019010309072101).

### 2.1. Animals and Management

Twelve “Trotteurs Français” geldings (4–10 years old, 505 ± 35 kg BW) stabled in individual boxes bedded with straw in Lab To Field’s experimental stud (Créancey, France) were enrolled in the study. Horses were allowed 1.5 h free exercise daily in a sand arena and were trained six times a week in an automatic walker (1 h at 1.7 m/s). Vaccination against tetanus and influenza (ProteqFlu-Te, Merial, Lyon, France) and deworming (Eraquell, Virbac, Carros, France) were verified before the start of the trial. The health status of horses and their dietary intakes were followed daily during the experimental trial. A thorough clinical examination was performed daily during the dietary stress only. The clinical variables included the measurement of both heart and respiratory rates, the rectal temperature, the observation of the fecal consistency, and abdominal pain. Horses were also weighed and their body condition was evaluated every week.

### 2.2. Experimental Design, Diets and Supplementation

**Figure 1:**
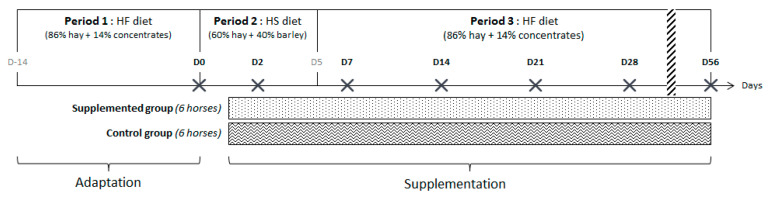
Diagram depicting the timing of the dietary changes from high-fiber (HF) to high-starch (HS) and vice versa, days (D) of sample collections (black cross), and the administration of feed supplement.

The twelve horses were separated in two homogeneous groups in terms of age and weight (supplemented = group S and control = group C) and enrolled in a longitudinal study of 10 weeks divided into 3 periods, each associated with a diet. Both diets were offered in two equal meals daily (at 08:15 and 16:45). During the first period (2 weeks, day (D) –14 to D0), horses were fed with a HF diet. During the second period (5 days, D0–D5), they were switched abruptly to a HS diet starting at D0 with the afternoon meal. During the third period (7 weeks, D5–D56) they returned to the HF diet without any dietary transition with the afternoon meal at D5 (Figure 1). The HF and HS diets were formulated to be isoenergetic and to meet 100% of the energy requirements for a horse subjected to moderate work [15]. Each horse had free access to a NaCl block and to an automatic waterer. The HF diet was composed of 86% dry matter (DM) of hay and 14% DM of pellet concentrates (Base 3, SERKO, Gemmelaincourt, France). The HS diet was based on 60% DM of hay and 40% DM of rolled barley. HF and HS diets provided 69.5 g and 217.1 g starch/meal/100 kg BW, respectively (Table 1). With the HS diet, starch intake was above the maximum recommendation per meal for hindgut ecosystem health [16], and the daily forage intake was less than the lowest recommended limit [17].

Group S horses were supplemented from D1 to D56 with a daily 3 g dose of FLORÉQUILIBRE CVX (Wamine, Paris, France) containing a blend of three LAB, *Lactobacillus acidophilus* LA201, *Ligilactobacillus salivarius* LA302, and *Bifidobacterium lactis* LA304 at 1.6 × 10^10^ colony forming units (CFU)/g (Biocéane, Le Havre, France). The supplement was incorporated into a mixture of 100 g alfalfa rehydrated with water and distributed each morning immediately before the morning meal. Horses from the C group were fed the same mixture without the supplement.

### 2.3. Sample Collection

Fecal samples of all horses were collected on average 5 h after the morning meal as follows: at the end of the first HF period before the diet change, as the baseline point (D0), 43 h after the first abrupt dietary change from HF to HS during the second period (D2), 43 h after the second abrupt change from HS to HF during the third period (D7), and then once per week (D14, 21, 28, and 56) during the third period to follow the evolution after the second abrupt dietary change (Figure 1).

Fecal samples were subsampled sterilely for molecular biology analysis and stored at –80 °C. In parallel, an aliquot of each fecal sample was placed in a covered flask filled to the maximum capacity to avoid the presence of oxygen and placed at 38 °C during transportation. Then, bacterial cultures were inoculated under a continuous flow of CO_2_ at our microbiology laboratory. Another aliquot was sampled to enumerate protozoa. Fecal samples were placed at 65° C for 72 h to measure the DM content of the samples. The rest of the solid fecal sample was filtered through a 100 µm diameter mesh, and aliquots of the filtrate were immediately frozen (−20 °C) after being sub-sampled for lactic acid and VFAs analyses (in a preservative solution composed of 4.25% H_3_PO_4_ and 1.0% HgCl_2_). The acidity of a filtered fecal sample was recorded with an electronic pH meter (CyberScan pH 510; Eutech Instrument Europe B.V., Landsmeer, The Netherlands) immediately after sampling.

### 2.4. DNA Extraction and Sequencing of the V3–V4 Region of the 16S rRNA Gene

Total DNA was extracted from 0.25 g fecal content as described by Yu and Morrison [18]. The quantity and the purity of obtained DNA were assessed by spectrophotometry (Eppendorf, Hamburg, Germany). The V3–V4 region of the 16S rRNA gene was amplified, sequenced, and analyzed as previously described by Grimm et al. [19]. Briefly, the V3–V4 region of the 16S rRNA gene was amplified by a first round of polymerase chain reaction (PCR) and checked by electrophoresis on a 2% agarose gel. PCR products were then used to perform the sequencing analysis (Genotoul bioinformatics platform, Toulouse, France). Amplicons were purified and submitted to a second round of PCR and the resulting products were sequenced using an Illumina MiSeq run of 250 base paired ends according to the manufacturer’s instructions (Illumina Inc., San Diego, CA, USA). All raw sequences obtained during this study were submitted to the NCBI Sequence Read Archive (accession number PRJNA723498).

### 2.5. Sequence Processing and Data Analysis

Bioinformatics analysis was performed using the Find Rapidly operational taxonomic unit (OTU) with Galaxy Solution (FROGS pipeline). Sequences were filtered, chimeric DNA sequences were removed using VSEARCH, and reads were clustered into operational taxonomic unit (OTUs) with Swarm. Resulting OTUs that were not present in at least 2 samples or whose abundance was <5 × 10^−5^, were then aligned to the SILVA 123 database [20,21] using basic local alignment search tool (BLAST). Relative abundances of the different families and genera were calculated and expressed as the percent of the total number of sequences. The within-sample (α) diversity was estimated using Shannon and InvSimpson indexes. The between-samples (β) diversity was estimated by computing unweighted or weighted UniFrac distances and was visualized with principal coordinate analysis (PCoA). Visualization with a double PCoA (DPCoA) was used to plot in the same space phylogenetic distances between communities (dissimilarity matrix) and species distribution among communities (abundance or presence/absence matrix) [22].

Linear discriminant analysis effect size (LEfSe) was performed using the Galaxy software package from the Huttenhower lab [23] to determine significant taxonomic differences between the fecal microbiota of control and supplemented groups. The factorial Kruskal–Wallis sum-rank non-parametric test (α = 0.05) was used to identify taxa with significant differences in abundance between groups (using all-against-all comparisons). Linear discriminant analysis (LDA) was performed to estimate the effect size of each differentially abundant feature based on a threshold of a logarithmic LDA score of three. Significant taxa were used to generate taxonomic cladograms illustrating differences between the fecal microbiota of control and supplemented groups.

### 2.6. Clostridioides difficile Quantification

Quantitative PCR (qPCR) was performed to measure *Clostridioides difficile*, formerly *Clostridium difficile* [24], in fecal DNA extracts as described by Bandelj et al. [25]. Oligonucleotide primers CDIFF16S-F (5’-TTGAGCGATTTACTTCGGTAAAGA-3’) and CDIFF16S-R (5’-TGTACTGGCTCACCTTTGATATTCA-3’), and CDIFF16S TaqMan probe (FAM-CCACGCGTTACTCACCCGTCCG) (MGW, Eurofins Genomics, Ebersberg, Germany) specific for the *C. difficile* 16S rRNA gene (accession number: AB548672) were used for the qPCR assay [26]. The qPCR mixture (final volume 25 µL) was composed of FastStart Universal Probe Master Mix 2× (12.5 μL) (Merck KGaA, Darmstadt, Germany), primers (900 nM), probe (200 nM), and DNA (1 µL). Twenty nanograms of bovine serum albumin (BSA) were added to the mixture to reduce the effect of inhibitors. The qPCRs were performed in a 96-well plate format using a CFX96 Real-Time System (Thermo Fisher Scientific, Indianapolis, IN, USA). The thermal cycling conditions used were described by Bandelj et al. [25]. One negative control was included to verify any cross-contamination. An internal positive standard was used that consisted of 1 ng/μL of *C. difficile* ATCC 9689 (DSMZ, Leibniz Institute, Berlin, Germany). Results were expressed in cells of *C. difficile* per mL, based on a standard curve obtained from serial dilution of the positive standard. The slopes of the calibration lines were between −3.21 and −3.48 (r2 = 0.99).

### 2.7. Protozoa Concentration Analysis

Fecal samples were suspended in four volumes (1:4 *v*/*v*) of formalin (18.5%) [27]. Lugol solution was added to 1 mL of the mixture for at least 15 min before enumeration [28] using a Thoma cell counting chamber under an optical microscope. Protozoa concentrations expressed in number of cells/mL were converted into base-ten logarithms.

### 2.8. Analysis of Functional Bacterial Groups

Enumeration of bacteria from fecal samples was performed using conventional anaerobic culture techniques in roll tubes [29]. To maintain strict anaerobic conditions, fresh fecal samples were diluted 10-fold in a mineral solution under a continuous flow of CO_2_ [30]. Samples were then inoculated on non-selective medium and on selective media containing starch and lactic acid, for enumeration of total anaerobic bacteria, amylolytic and lactic acid-utilizing bacteria, respectively [31], after 48 h at 38 °C. The cellulolytic bacteria were cultured anaerobically in roll tubes containing a complex liquid medium with one filter paper strip as cellulose source for 14 days at 38 °C [32]. Bacteria concentrations were determined by colony enumeration (CFU—colony forming units) or using the McGrady method of the most probable number, solely for cellulolytic bacteria [32]. Finally, all bacterial concentrations were converted into base-ten logarithms.

### 2.9. Determination of Fermentation End-Products

Total VFAs, acetic (C2), propionic (C3), isobutyric (iC4), butyric (C4), isovaleric (iC5), and valeric (C5) acid concentrations were assayed by gas–liquid chromatography (Clarus 500; PerkinElmer, Courtaboeuf, France) [33]. Each VFA concentration was expressed in mmol/L of fecal filtrate and as a proportion of the total VFA concentration, and the ratio ((C2+C4)/C3) was calculated as described by Sauvant et al. [34]). D-lactic acid and L-lactic acid concentrations were measured spectrophotometrically at 340 nm (MRX revelation, Dynatech Laboratories. Guyancourt, France) using an enzymatic colorimetric method (Megazyme, D-/L-lactic acid (D-/L-lactic acid) (Rapid) Assay Kit, Megazyme International Ireland Ltd., Wicklow, Ireland) as described by Grimm et al. [31]. Lactic acid concentrations were expressed in mmol/L of fecal filtrate and as a proportion of the total lactic acid concentration.

### 2.10. Statistical Analysis

Data were statistically analyzed using the MIXED procedure in SAS v 9.3 (SAS Inst, Inc., Cary, NC, USA) with a model including supplementation, day, and the interaction between supplementation and day as fixed effects. Individual horse values were included as intercepts and as a random effect to avoid baseline differences because of the longitudinal design of the trial. Days were considered as repeated measures with the horse as subject. In case of significant day × supplementation interactions, means have been separated using the pdiff option in the least squares means statement. The significance threshold was set at *p* < 0.05, and trends were considered at *p* < 0.10.

## 3. Results

### 3.1. Clinical Follow-Up of Horses

No signs of colic were observed during the experimental trial and horses were healthy. Their heart and respiratory rates were in normal ranges and the rectal temperature was below 38 °C over time. Loose feces were observed between D1 and D5 at least one time for ten horses in twelve, but horses did not display any sign of abdominal discomfort or pain. No feed refusals were noticed. However, two control horses and two supplemented horses took longer times (1–4 h) to consume their rolled barley meal entirely at D1, D2, D3, D4, and D5. No differences were observed between groups C and S.

### 3.2. Analysis of Bacterial 16S rRNA Gene Sequencing

#### 3.2.1. Sequencing Metrics

A total of 2,086,353 sequences were obtained from the 83 fecal samples. The fecal sample collected at D28 from one supplemented horse was excluded from the analysis because sequencing of this sample failed. After quality control, an average count of 13,090 ± 3227 (range: 7879–25,194) reads was obtained per sample. The sequences were clustered into 2039 OTUs, with a mean of 1256 ± 123 (range: 957–1583) OTUs per sample. OTUs were assigned into 9 phyla, 14 classes, 22 orders, 45 families, and 119 genera. In terms of relative abundance, 29% of genera and 98% of species remained unknown.

#### 3.2.2. Richness and Diversity

**Figure 2:**
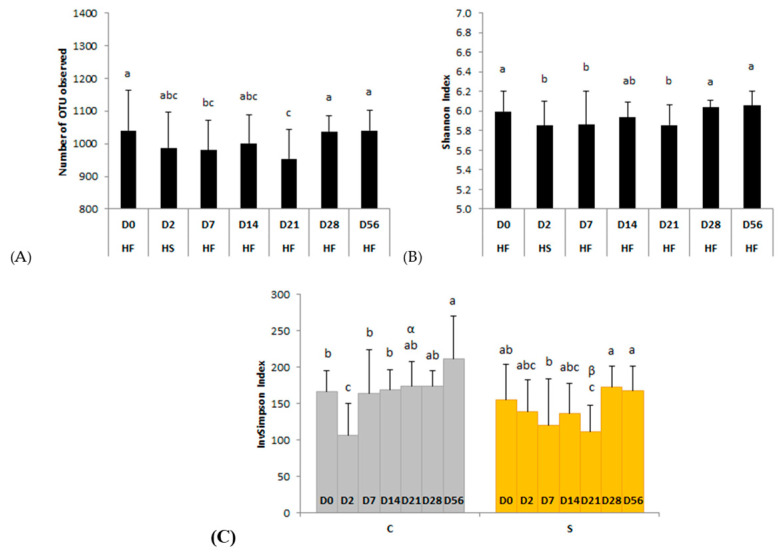
Modifications of the bacterial diversity and richness assessed by (**A**) the number of observed operational taxonomic units (OTUs), (**B**) Shannon, and (**C**) InvSimpson indexes during two consecutive dietary changes from high-fiber (HF) to high-starch (HS) diet and vice versa. On plots (**A**,**B**), means with different superscripts differ at *p* < 0.05 for each day. On plot (**C**), different Latin superscripts (a, b, c) represent significant variations between days for each supplementation whereas different Greek superscripts (α, β) describe significant variations between supplementations for each day. Error bars are based on standard deviations for each day (D).

We observed no day × supplementation interaction for the number of observed OTUs or Shannon index. The number of observed OTUs was significantly (*p* = 0.0328) lower than the basal state (D0; 1040 ± 125 observed OTUs) two days after the second dietary change from HS to HF (D7; 981 ± 92 observed OTUs) and returned to the initial value 3 weeks after the changes (D28; 1038 ± 49 observed OTUs) (Figure 2A). The Shannon index was significantly (*p* = 0.0046) lower than D0 two days after both dietary changes (D2, D7) and returned to basal value three weeks after the changes (from D28) (Figure 2B).

When the InvSimpson index, influenced by the dominance/abundance of OTUs, was considered, a significant day × supplementation interaction was observed (*p* = 0.0260) (Figure 2C). This index was significantly lower at D2, two days after the change from HF to HS, compared with the basal state (D0) in the feces of control horses. In supplemented horses it was significantly lower on D21 compared with D0, D7, D28, and D56. On D21, the InvSimpson index was significantly lower with supplementation than without (*p* = 0.0179).

#### 3.2.3. Community Structure

**Figure 3:**
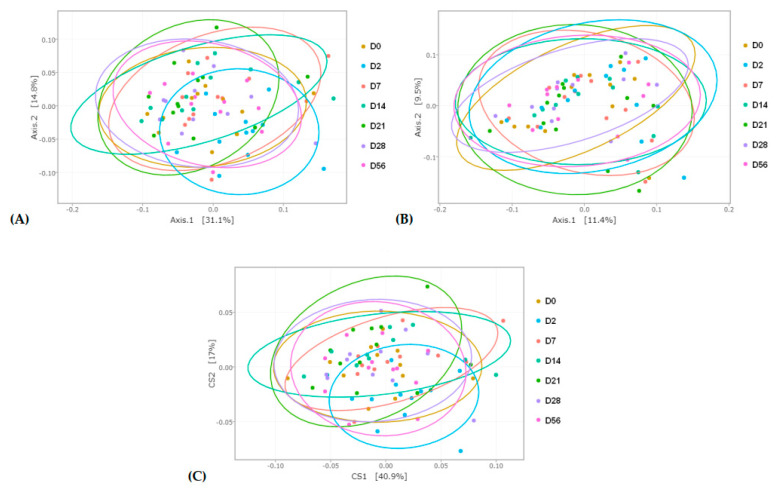
Principle coordinate analysis (PCoA) representing day (D)-related modifications of the bacterial membership and community structure in the feces of horses assessed by (**A**) weighted and (**B**) unweighted Unifrac distances. (**C**) Double PCoA (DPCoA) representing day-related modifications of the bacterial community structure and diversity assessed using abundance and phylogenetic components.

Weighted and unweighted UniFrac principal coordinate analysis (PCoA) and double PCoA (DPCoA) were used to compare community structure among samples during the seven days of sampling (Figure 3A–C). With both types of analysis, a smaller distance between samples indicates more closely connected communities. PCoA based on weighted UniFrac distances and DPCoA revealed a modified community structure two days after the abrupt change from HF to HS (D2). The distance between the other days appeared similar. Unweighted UniFrac distances did not allow any discrimination between the days.

#### 3.2.4. Bacterial Population Composition

**Figure 4:**
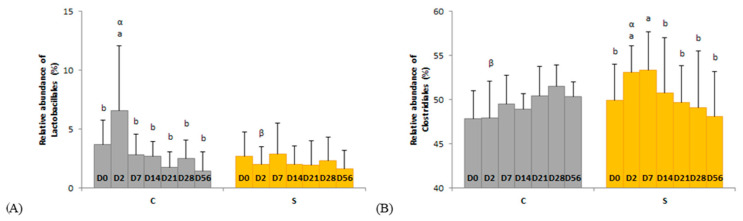
Day × supplementation interactions for two orders: (**A**) Lactobacillales and (**B**) Clostridiales. For each plot, different Latin superscripts (a, b) indicate significant variations between days for each supplementation. Different Greek superscripts (α, β) described significant variations between supplementations for each day. The two modalities of supplementation are control (C) and supplemented (S) groups. Error bars are based on standard deviations for each day (D).

**Figure 5:**
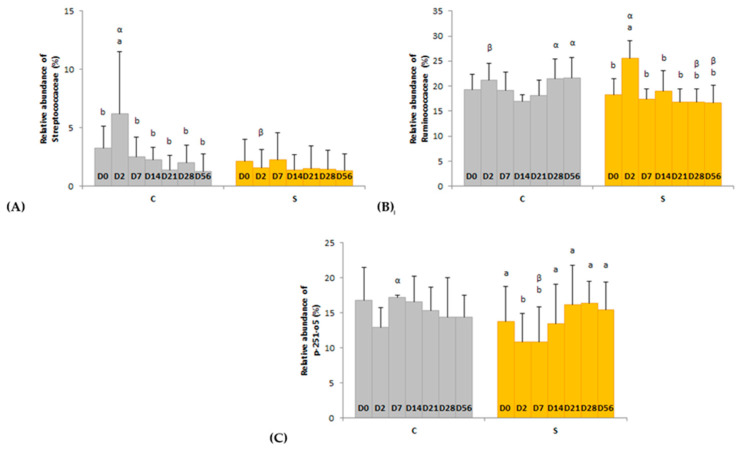
Day × supplementation interactions for three families: (**A**) Streptococcaceae, (**B**) Ruminococcaceae and (**C**) p-251-o5. For each plot, different Latin superscripts (a, b) indicate significant variations between days for each supplementation. Different Greek superscripts (α, β) described significant variations between supplementations for each day. The two modalities of supplementation are control (C) and supplemented (S) groups. Error bars are based on standard deviations for each day (D).

Several day × supplementation interactions were found at different taxonomic levels. At the order and family levels, two and three significant interactions were observed, respectively (Figure 4 and Figure 5). In group C only, the relative abundances of Lactobacillales (*p* = 0.0326) and Streptococcaceae (*p* = 0.0245) were higher two days after the first dietary change from HF to HS (D2) than in the basal state (D0) and returned to initial levels two days after the second dietary change from HS to HF (D7). The relative abundances of Clostridiales and p-251-o5 in group S were respectively higher (*p* = 0.0080) and lower (*p* = 0.0212) two days after both dietary changes (D2, D7) compared with the basal state (D0) and returned to initial values at D14. The relative abundance of Ruminococcaceae was higher two days after the first dietary change in group S only (*p* = 0.0016). At genus level, the relative abundance of three and eight taxa were significantly modified in groups C and S, respectively (Table S1).

The enrichment in the relative abundance of bacterial taxa in the equine fecal microbiome was investigated by LEfSe analysis based on the day × supplementation interactions. LEfSe revealed nine and ten known taxa associated with groups C and S, respectively (Table 2).

**Figure 6:**
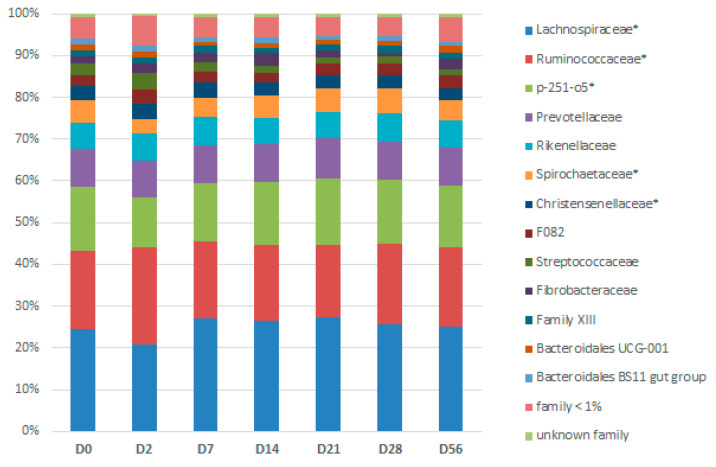
Days (D)-related bacterial modifications at the family level during two consecutive dietary changes from high-fiber (HF) to high-starch (HS) diet and vice versa. The relative abundance of families with star superscripts (*) differed significantly between days.

At phylum level, the relative abundance of Firmicutes (*p* = 0.0254), Proteobacteria, and its lower phylogenetic rank Enterobacteriaceae (*p* = 0.0002), were highest two days after the first dietary change from HF to HS (D2) (Table S2). By contrast, the relative abundance of Spirochaetes and its lower phylogenetic ranks Spirochaetaceae and Treponema 2 (*p* = 0.0002) was significantly the lowest at the same time (D2). The relative abundance of seven families and eighteen genera were also significantly modified according to the day (Figure 6 and Table S2).

### 3.3. Clostridioides difficile Quantification

The concentrations of *C. difficile* cells were lower than the limit of quantification of 2.25 × 10^5^ cells/µL in all samples.

### 3.4. Protozoa Concentration Analysis

We found no significant interactions, day or supplementation effects, on fecal protozoa concentrations. The mean concentration was 2.47 ± 1.22 × 10^5^ cells/mL.

### 3.5. Analysis of Bacterial Functional Groups

**Figure 7:**
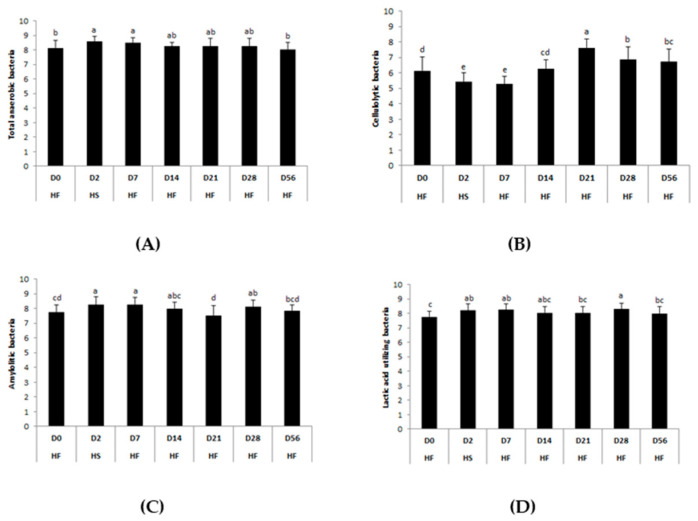
Days–related modifications of the bacterial functional groups’ (**A**) total anaerobes, (**B**) cellulolytics, (**C**) amylolytics, and (**D**) lactic acid utilizing during two consecutive dietary changes from high-fiber (HF) to high-starch (HS) diet and vice versa. Bacterial concentrations are expressed in log10 colony forming units /g feces. For each plot, means with different superscripts (a, b, c, d) differ at *p* < 0.05 for each day. Error bars are based on standard deviations for each day (D).

No significant interaction or supplementation effect was found for the functional groups. A significant effect of the day was observed for all bacterial groups (Figure 7).

Total anaerobic (*p* = 0.0401), lactic acid-utilizing (*p* = 0.0044), and amylolytic (*p* = 0.0004) bacteria concentrations increased significantly two days after the first abrupt dietary change from HF to HS (D2) compared with the basal state (D0). A significant fall in cellulolytic bacteria concentration was observed at the same time (*p* < 0.0001). After the second abrupt dietary change from HS to HF, nine days (D14) was required for bacterial concentrations to return to basal levels. The concentration of cellulolytic bacteria peaked two weeks after the second dietary change (D21) and was still higher than initial values at the end of the trial (D56). Lactic acid-utilizing and amylolytic bacteria concentrations significantly increased three weeks after the second dietary change (D28) and reached basal values four weeks later (D56).

### 3.6. Microbial Activity

No significant interactions or effects of the supplementation were observed regarding the parameters of the microbial activity, but a significant effect of time was found for some fecal parameters (Table 3).

Fecal DM increased significantly from the beginning of the trial (D0) until more than two weeks after the dietary stress (D21) (*p* < 0.0001). At the end of the trial (D56), fecal DM was still higher than basal levels. The fecal pH also varied over time (*p* = 0.0081), significantly decreasing during the dietary stress (D2), returning to basal values 2–9 days after the return to a HF diet (D7–D14), and decreasing again 1 week later (D21).

The proportion of acetic acid significantly decreased (*p* = 0.0023) whereas the proportion of valeric acid significantly increased (*p* = 0.0023) during the dietary stress (D2) compared with basal proportions (D0). Values returned to basal proportions at D7, two days after the second dietary change from HS to HF. L-lactic acid proportion reached the highest values one week after the second dietary shift (D7) while the D-lactic acid proportion was significantly lower (*p* = 0.0449) than the basal state (D0). Proportions of both lactic acids returned to initial values one week later (D14).

No significant modifications were observed for total VFAs and lactic acid concentrations (Figure S1).

## 4. Discussion

This study had three aims. First, to observe whether bacterial modifications occurred in feces after an abrupt change from a HF to a HS diet as previously reported for the hindgut contents. Second, we monitored variations in the microbiota after the return to the initial HF diet. We put a special emphasis on cellulolytic bacteria and fibrolytic activity. Finally, we explored whether supplementation with a blend of *L. acidophilus*, *L. salivarius*, and *B. lactis* could support the stability of the hindgut microbiota of horses subjected to these two consecutive dietary stresses.

### 4.1. A Sudden Change from HF to HS Alters the Diversity, Structure and Activity of the Ecosystem (D0–D2)

We observed a decrease in bacterial α-diversity and modifications of β-diversity in the feces 43 h after the abrupt change from a HF to a HS diet (D2), similar to previous findings for equine cecal contents that measured alterations in the first 12 h after the first meal of concentrate-rich diets [9]. Alteration in α-diversity, as estimated by the Shannon index, was more influenced by species richness than species evenness [35]. The abrupt incorporation of HS meals caused an alteration of the community structure, but not of the community membership, and revealed a fundamental reorganization of the bacterial population. Indeed, we observed an increase in the relative abundance of probable NFC utilizers (*Prevotellaceae UCG-004*, *Ruminococcaceae UCG-005*, and *Ruminococcaceae UCG-014*). The relative abundance of the starch-utilizer Lactobacillales and its lower taxonomic levels (Streptococcaceae and *Streptococcus*) also increased in the feces of control horses, as was observed in the cecum by Warzecha et al. [9] in the first 12 h after the first meal of concentrate-rich diets. In fact, the concentration of amylolytic bacteria increased when our horses abruptly received a mean of 2 kg of rolled barley/meal. This was consistent with the increased concentrations in *Streptococci* and *Lactobacilli* enumerated in cecal and colonic contents of horses abruptly fed rolled barley as 30–50% of their dietary intake [6] or 2.25 kg per meal [7]. The HS diet was formulated to exceed 200 g starch/100 kg BW/meal to surpass the maximal prececal digestion of starch, leading to starch flow in the hindgut [16] and potentially causing alteration of the hindgut ecosystem. As previously reported for the hindgut, we found that the abrupt incorporation of rolled barley in meals led to an increase in total lactic acid concentration in the feces (*p* = 0.1342), particularly that of L-lactic acid (*p* = 0.1090), because bacterial starch digestion is associated with significant acidification [6,7]. Consistent with the lactic acid production, we observed an increase in lactic-acid utilizing bacteria and an increase of the relative abundance of Veillonellaceae (*p* = 0.1153), as was also measured in equine cecal contents in the first 12 h after a first high-concentrate meal [9]. Thus, the intensive starch digestion in the hindgut induced acidosis in feces, beginning 43 h after the first HS meal.

This hindgut acidification subsequently affected bacterial fibrolytic bacteria and activity. In our study, the lower fecal pH induced a subsequent fall in concentrations of cellulolytic bacteria, which are known to be very sensitive to acidity [36]. A numerical decrease in cellulolytic bacteria was also reported in the RVC 29 h after the abrupt dietary abrupt [6]. A decrease in xylanolytic and pectinolytic bacteria concentrations was also observed in the cecal content of a pony abruptly fed 100% ground corn [5]. Concomitant with the fall in cellulolytic bacteria concentrations, our sequencing analysis revealed a decrease in the relative abundances of several potentially fibrolytic taxa, notably Lachnospiraceae (*Lachnospiraceae ND3007 group*, *Lachnospiraceae NK4A136 group*, *Lachnospiraceae UCG-008*) [37]. The relative abundance of several fiber degraders such as the *Eubacterium ruminantium group* [38], the recently defined *Agathobacter* [39,40], and *Roseburia* [41,42] also fell at D2. The relative abundances of Spirochaetes and its lower phylogenetic levels dropped too. Spirochaetes members were identified as primary agents of xylan degradation in termites [43]. In horses, Spirochaetes is recognized as a dominant phylum that belongs to the core microbiome [44] but its role remains unknown. However, an OTU of Spirochaetes was found to be lower when horses were fed a HS diet [45]. Thus, members of Spirochaetes may be sensitive to dietary substrates, especially fibers, and may play a role in fibrolysis. Because of these modifications in bacteria, the fecal proportions of several VFAs were modified, but their concentrations did not vary significantly. Low acetic acid and high valeric acid proportions were probably attributable to intense amylolytic activity and lower fibrolytic activity. Indeed, acetic acid is the main VFA produced from fiber fermentation, whereas valeric acid is an end-product of lactic acid utilizers [3]. Several taxa took advantage of the decrease of the relative abundance of fibrolytic bacteria: we found increased relative abundances of Proteobacteria and Enterobacteriaceae, as described for cecal contents by Warzecha et al. [9] during the first 12 h following a dietary change. Many enteropathogenic bacteria, such as *Salmonella* spp., belong to the Proteobacteria phylum. In humans, an increase in Proteobacteria populations is considered a bacterial signature of dysbiosis [46]. Infections with the pathogenic bacteria *Salmonella* spp. and *C. difficile* are major factors in the origin of equine acute diarrhea [47]. However, we did not observe modification to fecal concentrations of *C. difficile*.

The overall bacterial changes in the feces of horses 43 h after the abrupt incorporation of HS meals represented a typical picture of dysbiosis [48]. At present, the equine hindgut microbiota is usually examined using feces because access to cecal and colonic contents is restricted to the use of fistulated horses. Although fecal contents are not representative of the hindgut in terms of bacterial population structure [49]. The variations in cecal, colonic, and fecal microbiota under dietary change may be similar [31]. Investigating feces 43 h following the first HS meal is close to observing the cecal and colonic contents in the first 16 h after a dietary change, respectively, depending on individuals’ transit rate. Indeed, the transit time of digesta in the hindgut has been estimated at 30.6 h on average with a variation of 8 h [50]. Our data confirmed modifications in feces around two days after the first HS meal similar to those previously reported directly in the hindgut contents as soon as 5 h after an abrupt dietary change. Thus, the bacterial alterations we observed at D2 were related to the detrimental effects of transition from HF to HS diet. The microbial alterations observed did not lead to colic as no symptoms were noticed, and horses stayed healthy along the experimental trial. Our HS diet was formulated to lightly exceed the maximal amount of starch per meal for hindgut ecosystem health [16]. Furthermore, our horses might not have displayed individual susceptibility to the development of colic.

### 4.2. A Reverse Abrupt Change from HS to HF Induces Short and Long Term Modifications of the Fecal Ecosystem (D7–D56)

In our study, the bacterial richness and diversity, respectively estimated by the number of OTUs and the Shannon index, decreased during the dietary stress, and remained lower than basal values two days after HF diet reintroduction. The disappearance of numerous OTUs persisting 16 days after refeeding horses with HF indicated that two consecutive and closely timed abrupt diet changes could be detrimental to the horse microbiota. More than three weeks were necessary after the reintroduction of the HF diet to definitively recover the bacterial richness and diversity.

The only study on the impacts of such a reverse diet change in a pony reported that bacterial functional groups returned to similar proportions to those observed before the first abrupt change by 1.5 weeks [5]. In the present study, nine days were required for the reestablishment of the hindgut bacterial functions. However, some alterations were still observed 3–7 weeks after the dietary stresses, suggesting the occurrence of “waves” in the ecosystem after a stressful event. The inversion of the proportions of D- and L-lactic acids with a predominance of L-lactic acid compared with the basal state that was observed two days after the end of the dietary stress was also noticed three weeks later and persisted at the end of the trial. In equines, D- and L-lactic acids are produced in different quantities depending on the strain of lactic acid-producing bacteria [51]. This change in lactic acid isoforms could be a marker of the shift in bacterial communities. Bacterial members and activity take time to change de novo to respond to the new dietary substrates provided by the diet and by the subsequent microbial metabolism. A week after the reverse diet change, the decrease in the proportion of fecal valeric acid could be consequent to variations in lactic acid production, and coincided with low concentrations of lactic acid utilizers [3]. The highest concentration of lactic acid utilizers three weeks after the return to a HF diet demonstrated the capacity of the microbiota to adapt to maintain homeostasis by decreasing hindgut acidity. Thus, lactic acid producers and utilizers probably remained impacted 1–2 months after the abrupt introduction of HS meals.

Concomitant with the alterations observed for NFC fermentation, changes in fibrolytic function occurred during the reverse abrupt change from HS to HF. In the short term, two days after the diet had returned to HF, concentrations of cellulolytic bacteria remained strongly altered. However, probable fiber degraders from the Lachnospiraceae family (*Lachnospiraceae NK4A136 group*, *Lachnospiraceae UCG-002*, *Lachnospiraceae UCG-008,* and *Lachnospiraceae UCG-009*) reached higher relative abundances than basal values two days after the second abrupt dietary change [37]. Furthermore, the relative abundance of *Agathobacter* and *Blautia* also peaked at this time and one week later, respectively. These fiber-degrading genera are also known to be butyric acid-producers [39,52] that contribute to intestinal homeostasis. Thus, the microbiota reorganized a week after the first dietary change from HF to HS to ensure fiber degradation. One week after the return to a HF diet, cellulolytic bacteria concentrations were similar to the basal state and even bloomed one week later to achieve their highest concentrations, as described two weeks after an antibiotic challenge [53]. Previous data indicated that the highest cellulolytic bacteria counts were found three weeks after the return to a HF diet [5]. Similar change in fibrolytic genera were observed 2–3 weeks after the second abrupt dietary change, with respect to the relative abundances of the Lachnospiraceae family, *Eubacterium ruminantium* group and *Eubacterium nodatum* group genera [37]. This bloom of cellulolytic bacteria coincided with a decrease in the fecal pH, and the highest numerical fecal concentration of VFAs and DM. This could suggest intense fiber fermentation leading to higher VFA production and mucosal absorption, resulting in higher water absorption in the hindgut and higher fecal DM [54]. In the longer term, cellulolytic bacteria concentrations still remained elevated two months after the reintroduction of a high amount of forage. Similarly, the relative abundances of two fibrolytic genera, *Eubacterium xylanophilum group* [38] and *Cellulosilyticum* [55], achieved maximal values at the end of the trial. Thus, fibrolytic members probably recolonized their ecological niches progressively after the dietary stresses to ensure the degradation of fiber from forage.

### 4.3. Effects of Dietary Supplementation with B. lactis, L. acidophilus and L. salivarius

Bacterial diversity significantly decreased two days after the abrupt change from a HF to a HS diet in control horses but not in supplemented horses, suggesting that bacterial probiotics conferred better resistance to the dietary stress. However, we did not observe an effect of the dietary supplementation on bacterial functional groups or fermentation end-products at any time around the two dietary changes. Our results are consistent with the limited effects observed on nutrient digestibility and on reducing the risk of acidosis that have been reported previously in horses fed *L. acidophilus* alone or in combination with other LAB, 13 days before and after an abrupt change from low-starch to a HS diet [13].

However, the 16S RNAr sequencing investigation revealed differences between supplemented and non-supplemented horses. The relative abundance of *Succinivibrio* was enriched when supplemented horses were fed a HS diet, which was associated with higher fecal D-lactic acid concentrations (*p* = 0.0880). *Succinivibrio* members are known to contribute to starch digestion in the rumen [56]. Our LAB blend might promote members of *Succinivibrio* to ensure fermentation of prececally undigested starch in the hindgut. Improved starch digestion could also be the direct consequence of LAB supplementation because higher relative abundances of Lactobacillaceae were found in supplemented horses during the trial (*p* = 0.0620). This was consistent with the higher concentrations of *Lactobacilli* in colonic contents of horses supplemented with LAB 6 h after a barley meal [12]. Another interesting trend was the higher fecal concentrations of protozoa in control horses two days after the first diet change (*p* = 0.0793). Protozoa contribute to reducing subacute acidosis by decreasing the amount of starch fermented in the rumen [57]. The elevated standard deviations observed two days after dietary stresses highlighted the individual variability of bacterial alteration after dietary changes and supplementation in terms of bacterial modifications and response times. This could partially explain the controversial results found after probiotic LAB supplementation in equines [58]. Despite individual variabilities, supplementing horses with *B. lactis*, *L. acidophilus*, and *L. salivarius* might support starch digestion when horses are abruptly fed a HS diet by promoting starch-utilizing taxa, recruiting other microorganisms and deploying additional strategies in the hindgut.

The abrupt introduction of cereal, and therefore of starch, in the feed negatively affected fiber degradation and commensal microorganisms. The relative abundances of genera that belong to the Ruminococcaceae family, such as the *Ruminococcaceae NK4A214 group*, *Ruminococcaceae UCG-002*, and *Ruminococcaceae UCG-010*, reached their highest values two days after the abrupt change from a HF to a HS diet only in supplemented horses. Because members of the Ruminococcaceae are usually associated with fiber degradation [37], this indicates that dietary supplementation might help to maintain fibrolytic members in the hindgut microbiome after an abrupt change from a HS to a HF diet. Supplemented horses also displayed enrichment of the relative abundances of Ruminococcaceae (*Ruminococcaceae UCG 011*) and Lachnospiraceae (*Lachnospiraceae FCS020 group*, *FD2005* and *Lachnospiraceae AC2044 group*) families two days to more than two weeks after the return to a HF diet. By contrast, enrichment in the relative abundances of Lachnospiraceae and Ruminococcaceae genera was observed around 1–2 weeks later in non-supplemented horses. However, we did not detect any differences in cellulolytic activity. Thus, supplementation with a *B. lactis*, *L. acidophilus*, and *L. salivarius* blend might promote the earlier colonization and the reestablishment of several fibrolytic bacteria in the hindgut without modifying the fibrolytic activity in horses subjected to two abrupt dietary changes. This finding is in contrast to those of previous studies that reported a numerical increase in cellulolytic bacteria concentrations in cecal and colonic contents of horses supplemented with LAB [10,12].

The relative abundance of *Streptococcus* was only modified in control horses two days after the first HS meal. The *Streptococcus* genus includes both commensal and pathogenic bacteria [59]. Moreover, despite their very low relative abundance (<0.1%), the *Campylobacter* genus [60] and its higher taxonomic ranks were over-represented in the fecal microbiome of control horses throughout all the trial. It has been demonstrated previously that supplementation with *L. acidophilus* and two yeasts reduced the relative amounts of two enteropathogenic bacteria, *Escherichia coli*, and *Clostridium perfringens*, in the feces of adult horses [61]. Unfortunately, we did not investigate these pathogenic bacteria in feces. The dietary supplementation tested might limit the establishment of such opportunistic pathogenic bacteria in intestinal ecological niches. Supplemented horses displayed limited features of dysbiosis compared with the control horses [48]. Supplementation with this LAB blend might help to prevent dysbiosis in horses subjected to an abrupt dietary change.

## 5. Conclusions

Our observations in feces were consistent with changes in the diversity, structure, composition, and activity of the microbiota reported previously in the hindgut after incorporation of high levels of starch in the diet and provided a complete picture of this dysbiosis. The concomitant alteration of bacterial diversity and fibrolytic activity, the increase in opportunistic pathogens, and the sub-acidosis following the abrupt incorporation of HS meals may lead to digestive problems. Consequently, horses may develop colic related to such short-term modifications in the hindgut microbiota. Several days to several weeks were required for the recovery of the fecal microbiota after the abrupt return to diet rich in forage. The fecal microbiome recovered earlier than the bacterial functions, and a bloom of fibrolytic members and activity was observed to ensure fiber degradation was observed long after the dietary modification. Interestingly, profound modifications were observed about three weeks after the dietary stress suggesting “waves” of change in the ecosystem after an initial stress. Thus, digestive perturbations could also occur several weeks after refeeding horses a HF diet because of the delayed recovery of bacterial digestive functions. Supplementation with *B. lactis*, *L. acidophilus*, and *L. salivarius* might support starch degradation during the HS diet and help an earlier recolonization of fibrolytic members after the return to a HF diet. Supplementing horses with these three probiotic LABs might be an interesting strategy to support the equine hindgut microbiota when horses must be subjected to abrupt dietary changes.

## Figures and Tables

**Table 1:** Chemical and nutritional composition of the diets.

Diet	High-Fiber (HF)	High-Starch (HS)
Hay (% DM)	86	60
Concentrate (% DM)	14	-
Barley (% DM)	-	40
Digestive energy (kcal/100 kg BW)	4660	4660
Dry matter (DM) (g/100 g)	93.50	90.60
Hay ingestion level (% BW DM/day)	1.85	1.04
Pellet ingestion level (% BW DM/day)	0.30	-
Rolled barley ingestion level (% BW DM/day)	-	0.70
Starch ingestion level (% BW DM/day)	0.14	0.39
Crude protein (CP, % DM)	8.51	9.21
Neutral detergent fiber (NDF, % DM)	57.08	42.56
Acid detergent fiber (ADF, % DM)	34.31	24.77
Acid detergent lignin (ADL, % DM)	5.72	4.22
Starch (% DM)	6.59	22.19

The composition of both diets is expressed as a percentage of dry matter (DM) and the levels of ingestion are reported as a percentage of body weight (BW) DM per day.

**Table 2:** Linear discriminant analysis size effect (LEfSe) showing significant taxa modifications in the fecal microbiome of horses from control (C) and supplemented (S) groups.

Phylum > Class > Order > Family > Genus > *Species*	Day	LDA	*p*-Value
**Group C**			
Firmicutes > Clostridia > Clostridiales > Lachnospiraceae > Blautia	D7	4.275	0.030
Proteobacteria > Deltaproteobacteria > Desulfovibrionales > Desulfovibrionaceae > Mailhella	D14	3.991	0.025
Firmicutes > Clostridia > Clostridiales > Lachnospiraceae	D21	5.018	0.033
Firmicutes > Clostridia > Clostridiales > Lachnospiraceae > Lachnoclostridium	D21	4.391	0.039
Firmicutes > Clostridia > Clostridiales > Lachnospiraceae > Lachnoclostridium > *Clostridium asparagiforme*	D21	4.391	0.001
Firmicutes > Clostridia > Clostridiales > Ruminococcaceae > Eubacterium coprostanoligenes group	D28	4.042	0.028
Firmicutes > Clostridia	D56	5.161	0.034
Firmicutes > Clostridia > Clostridiales	D56	5.161	0.034
Firmicutes > Clostridia > Clostridiales > Ruminococcaceae > Pygmaiobacter	D56	4.099	0.014
Firmicutes > Clostridia > Clostridiales > Lachnospiraceae > Moryella	D56	4.310	0.044
**Group S**			
Proteobacteria	D2	5.038	0.006
Proteobacteria > Gammaproteobacteria	D2	5.015	0.002
Proteobacteria > Gammaproteobacteria > Aeromonadales	D2	5.025	0.001
Proteobacteria > Gammaproteobacteria > Aeromonadales > Succinivibrionaceae	D2	5.025	0.001
Proteobacteria > Gammaproteobacteria > Aeromonadales > Succinivibrionaceae > Succinivibrio	D2	5.025	0.001
Firmicutes > Clostridia > Clostridiales > Ruminococcaceae > Ruminococcaceae UCG-011	D7	3.924	0.044
Firmicutes > Clostridia > Clostridiales > Lachnospiraceae > Lachnospiraceae FCS020 group	D7	4.277	0.027
Firmicutes > Clostridia > Clostridiales > Lachnospiraceae > FD2005	D14	3.881	0.025
Firmicutes > Clostridia > Clostridiales > Lachnospiraceae > Lachnospiraceae AC2044 group	D21	3.853	0.036
Bacteroidetes > Bacteroidia > Bacteroidales > Bacteroidetes BD2 2	D28	4.169	0.026
Bacteroidetes > Bacteroidia > Bacteroidales > Bacteroidaceae	D28	4.006	0.017
Bacteroidetes > Bacteroidia > Bacteroidales > Bacteroidaceae > Bacteroides	D28	3.988	0.017
Spirochaetes > Spirochaetia > Spirochaetales > Spirochaetaceae > Treponema 2	D28	4.077	0.050
Spirochaetes > Spirochaetia > Spirochaetales > Spirochaetaceae > Treponema 2 > *Treponema pectinovorum*	D28	3.537	0.016

**Table 3:** Effect of the day (D) on the parameters of microbial activity in the feces during two consecutive dietary changes from high-fiber (HF) to high-starch (HS) diet and vice versa.

Days	D0	D2	D7	D14	D21	D28	D56	Mean ± Standard Deviation	*p*-Values		
Diets	HF	HS	HF	HF	HF	HF	HF		D	S	D × S
**Dry matter and pH in feces**											
Dry matter (%)	21.04 ^d^	21.16 ^cd^	22.16 ^bc^	22.65 ^ab^	23.47 ^a^	21.22 ^cd^	22.33 ^b^	22.00 ± 1.80	**<0.0001**	0.7909	0.2532
pH	6.80 ^ab^	6.63 ^cd^	6.77 ^abcd^	6.74 ^abc^	6.60 ^d^	6.92 ^a^	6.77 ^abc^	6.74 ± 0.27	**0.0081**	0.3168	0.7490
**Volatile Fatty Acid (VFA) concentration and proportion in feces**											
Total VFAs (mmol/L)	30.77	33.56	33.28	34.92	34.57	29.74	31.65	32.64 ± 10.04	0.7345	0.4071	0.1641
C2 (%)	67.56 ^a^	65.15 ^b^	69.01 ^a^	67.80 ^a^	68.06 ^a^	68.43 ^a^	68.62 ^a^	67.80 ± 3.40	**0.0023**	0.9080	0.7573
C3 (%)	20.60	21.20	19.29	20.71	20.48	19.67	20.53	20.36 ± 3.06	0.9513	0.5686	0.4974
iC4 (%)	1.94	2.45	2.01	1.84	1.88	2.00	1.76	1.98 ± 0.68	0.3142	0.6917	0.493
C4 (%)	6.75	7.50	6.53	6.85	6.68	6.83	6.45	6.80 ± 0.95	0.306	0.6203	0.4372
iC5 (%)	0.93	1.17	0.96	0.81	0.92	0.93	0.79	2.13 ± 0.72	0.1885	0.6171	0.4108
C5 (%)	2.22 ^bc^	2.53 ^a^	2.21 ^ab^	1.98 ^c^	1.97 ^bc^	2.14 ^bc^	1.85 ^c^	0.93 ± 0.28	**0.0023**	0.7236	0.096
(C2 + C4)/C3	3.71	3.61	4.02	3.69	3.74	3.94	3.77	3.78 ± 0.81	0.9305	0.6743	0.4984
**Lactic acid (LA) concentration and proportion in feces**											
Total LA (mmol/L)	0.98	1.08	1.08	0.92	1.08	0.92	1.17	1.03 ± 0.26	0.1342	0.4908	0.5077
D-LA (%)	51.28 ^a^	47.41 ^abc^	43.63 ^c^	49.38 ^ab^	46.66 ^abc^	45.35 ^bc^	45.69 ^bc^	51.28 ± 47.41	**0.0449**	0.4242	0.2612
L-LA (%)	48.72 ^c^	52.59 ^abc^	56.37 ^a^	50.62 ^bc^	53.34 ^abc^	54.65 ^ab^	54.31 ^ab^	48.72 ± 52.59	**0.0449**	0.4242	0.2612

For each line, means with different superscripts (a, b, c, d) differ at *p* < 0.05. Significant *p*-values are in bold.

## Data Availability

The data presented in this study are available in the Sequence Read Archive (SRA) of the National Center for Biotechnology Information (NCBI) [accession number PRJNA723498].

## Supplementary Materials

The following are available online at https://www.mdpi.com/article/10.3390/ani11051278/s1. Table S1: Effect time on the relative abundance of bacterial taxa at (A) phylum, (B) family, and (C) genus levels, Table S2: Interactions with the relative abundance of bacterial taxa at genus level, Figure S1: Days-related modifications of the proportions (%) and concentrations (mmol/L) of (A) lactic acids and (B) volatile fatty acids.
