# Supplementary material for: Sequential Modulation of the Equine Fecal Microbiota and Fibrolytic Capacity Following Two Consecutive Abrupt Dietary Changes and Bacterial Supplementation
Source: Animals (Basel). 2021 Apr 29;11(5):1278. doi: 10.3390/ani11051278 (PMC8144951; doi:10.3390/ani11051278)
Supplement: Supplementary file 1 [file animals-11-01278-s001.zip › animals-1186622-supplementary.pdf]

Table 1. Interactions with the relative abundance of bacterial taxa at genus level (abundance in percent. of the total number of sequences). Genus mean relative abundances are greater than 0.1% of the total relative abundance in all the fecal samples.

Interactions on genera	D0	D2	D7	D14	D21	D28	D56	Mean	SD	P-Value
	HF	HS	HF	HF	HF	HF	HF			
**Group C**										
Ruminiclostridium	0.28^{ab}	0.23^{b}	0.45^{a}	0.30^{b}	0.20^{b}	0.35^{b}	0.58^{a}	0.32	0.22	0.0147
Ruminococcaceae UCG-013	0.21^{b}	0.15^{b}	0.17^{b}	0.22^{b}	0.15^{b}	0.19^{b}	0.27^{a}	0.24	0.15	0.0064
Streptococcus	2.81^{b}	5.84^{a}	2.22^{b}	1.63^{b}	1.83^{b}	2.34^{b}	1.70^{b}	2.16	2.31	0.0245
**Group S**										
[Eubacterium] coprostanoligenes group	2.44^{bc}	3.24^{a}	2.48^{bc}	2.32^{b}	2.06^{c}	2.33^{c}	2.64^{bc}	2.52	0.78	0.0058
Alloprevotella	0.73^{bc}	0.95^{a}	0.62^{b}	0.79^{b}	0.47^{b}	0.63^{b}	0.58^{b}	0.56	0.34	0.0479
Candidatus Soleaferrea	0.33^{b}	0.46^{a}	0.36^{b}	0.23^{b}	0.24^{b}	0.35^{b}	0.35^{b}	0.35	0.24	0.0276
Lachnospiraceae UCG-004	0.21^{b}	0.12^{c}	0.29^{b}	0.25^{bc}	0.28^{b}	0.23^{ab}	0.32^{a}	0.27	0.16	0.0238
Rikenellaceae RC9 gut group	4.7^{b}	5.46^{a}	5.05^{a}	4.08^{b}	4.24^{b}	4.71^{b}	4.80^{b}	5.18	1.55	0.0233
Ruminococcaceae NK4A214 group	2.87^{bc}	3.61^{a}	3.35^{b}	2.51^{bc}	2.59^{bc}	2.64^{bc}	2.81^{c}	3.21	1.37	0.0032
Ruminococcaceae UCG-002	1.23^{b}	2.6^{a}	1.36^{b}	1.03^{b}	1.08^{b}	1.05^{b}	1.04^{b}	1.43	0.87	0.0054
Ruminococcaceae UCG-010	2.24^{abc}	2.83^{a}	2.38^{abc}	1.97^{b}	1.87^{c}	2.12^{bc}	2.78^{bc}	2.45	0.94	0.0261

For each line, means with different superscripts differ at $P < 0.05$.

Table 2. Effect time on the relative abundance of bacterial taxa at (A) phylum, (B) family and (C) genus levels (abundance in percent of the total number of sequences). Genus mean relative abundances are greater than 0.1% of the total relative abundance in all the fecal samples.

Effect of Days	D0	D2	D7	D14	D21	D28	D56	Mean	S.D.	*P*-Value
	HF	HS	HF	HF	HF	HF	HF			
**(A) Phylum**										
Firmicutes	52.43^{bc}	55.21^{a}	54.54^{ab}	52.54^{bc}	52.28^{bc}	53.32^{abc}	51.26^{c}	**53.08**	**3.81**	0.0254
Proteobacteria	0.83^{b}	1.91^{a}	0.86^{b}	0.81^{b}	0.70^{b}	0.68^{b}	0.85^{b}	**0.95**	**0.83**	0.0030
Spirochaetes	5.44^{ab}	3.46^{c}	4.65^{b}	5.54^{ab}	5.64^{ab}	5.98^{a}	4.88^{b}	**5.07**	**2.09**	0.0003
**(B) Family**										
BacteroidalesRF16group	0.83^{bc}	0.78^{c}	1.04^{b}	0.72^{c}	0.88^{bc}	0.83^{bc}	1.30^{a}	**0.91**	**0.36**	0.0002
Christensenellaceae	3.33^{abc}	3.77^{a}	3.72^{ab}	3.10^{c}	3.25^{bc}	3.31^{abc}	2.97^{c}	**3.35**	**0.82**	0.0145
CoriobacterialesIncertaeSedis	0.11^{ab}	0.10^{abc}	0.12^{a}	0.11^{ab}	0.07^{c}	0.10^{bc}	0.10^{abc}	**0.10**	**0.05**	0.0276
Lachnospiraceae	24.63^{b}	20.86^{c}	27.13^{a}	26.59^{ab}	27.25^{a}	25.51^{abc}	24.96^{ab}	**25.27**	**4.06**	<0.0001
Muribaculaceae	0.52^{b}	1.09^{a}	0.42^{b}	0.47^{b}	0.41^{b}	0.47^{b}	0.63^{b}	**0.57**	**0.51**	0.0035
Spirochaetaceae	5.28^{ab}	3.34^{c}	4.49^{b}	5.45^{ab}	5.48^{ab}	5.95^{a}	4.71^{b}	**4.94**	**2.11**	0.0002
Succinivibrionaceae	0.08^{b}	1.71^{a}	0.06^{b}	0.08^{b}	0.14^{b}	0.05^{b}	0.09^{b}	**0.28**	**0.77**	0.0001
**(C) Genus**										
[Eubacterium]nodatumgroup	0.13^{ab}	0.13^{ab}	0.10^{abc}	0.10^{abc}	0.07^{c}	0.14^{a}	0.09^{bc}	**0.23**	**0.06**	0.0244
[Eubacterium]ruminantiumgroup	0.41^{bc}	0.25^{d}	0.35^{bcd}	0.32^{cd}	0.57^{a}	0.48^{ab}	0.44^{abc}	**0.40**	**0.23**	0.0017
Agathobacter	1.02^{b}	0.77^{c}	1.17^{ab}	1.24^{a}	1.05^{b}	1.07^{b}	0.99^{b}	**1.04**	**0.30**	0.0003
Blautia	0.46^{bc}	0.36^{c}	0.60^{a}	0.50^{ab}	0.47^{ab}	0.48^{bc}	0.39^{bc}	**0.46**	**0.22**	0.0061

Celulosilyticum	0.12^{bc}	0.18^{ab}	0.13^{bc}	0.16^{b}	0.13^{bc}	0.09^{c}	0.23^{a}	**0.15**	**0.10**	0.0016
ChristensenelaceaeR-7group	3.30^{abc}	3.73^{a}	3.68^{ab}	3.06^{c}	3.22^{bc}	3.25^{abc}	2.94^{c}	**3.32**	**0.82**	0.0145
FamilyXIIIAD3011group	0.27^{bcd}	0.34^{ab}	0.31^{abc}	0.26^{cd}	0.22^{d}	0.37^{a}	0.26^{cd}	**0.29**	**0.10**	0.0029
LachnospiraceaeND3007group	0.37^{a}	0.24^{c}	0.27^{bc}	0.27^{bc}	0.26^{bc}	0.35^{ab}	0.28^{bc}	**0.29**	**0.14**	0.0237
LachnospiraceaeNK4A136group	3.98^{a}	2.98^{b}	3.73^{a}	4.26^{a}	4.17^{a}	3.66^{ab}	4.03^{a}	**3.85**	**1.23**	0.0202
LachnospiraceaeUCG-002	0.11^{abc}	0.10^{bc}	0.14^{a}	0.12^{ab}	0.12^{ab}	0.09^{c}	0.11^{bc}	**0.11**	**0.05**	0.0345
LachnospiraceaeUCG-008	0.52^{a}	0.40^{b}	0.58^{a}	0.54^{a}	0.53^{a}	0.60^{a}	0.52^{a}	**0.53**	**0.16**	0.0220
LachnospiraceaeUCG-009	2.52^{abcd}	2.07^{d}	2.75^{ab}	2.84^{a}	2.57^{abc}	2.23^{cd}	2.30^{bcd}	**2.48**	**0.72**	0.0278
PrevotelaceaeUCG-004	1.49^{bc}	1.91^{a}	1.45^{c}	1.54^{bc}	1.48^{bc}	1.80^{ab}	1.52^{bc}	**1.59**	**0.48**	0.0447
Roseburia	0.24^{ab}	0.12^{c}	0.22^{b}	0.23^{ab}	0.30^{a}	0.28^{ab}	0.25^{ab}	**0.23**	**0.13**	0.0017
RuminococcaceaeUCG-005	2.22^{b}	2.77^{a}	2.21^{b}	2.36^{b}	1.82^{c}	2.23^{b}	2.18^{b}	**2.25**	**0.65**	0.0002
RuminococcaceaeUCG-014	0.44^{b}	0.76^{a}	0.36^{b}	0.42^{b}	0.38^{b}	0.50^{b}	0.45^{b}	**0.48**	**0.26**	0.0003
Succinivibrio	0.10^{b}	1.35^{a}	0.06^{b}	0.11^{b}	0.12^{b}	0.07^{b}	0.12^{b}	**0.28**	**0.78**	0.0001
Treponema2	5.24^{ab}	3.31^{c}	4.46^{b}	5.41^{ab}	5.45^{ab}	5.91^{a}	4.65^{b}	**4.89**	**2.12**	0.0002

For each line, means with different superscripts differ at $P < 0.05$.

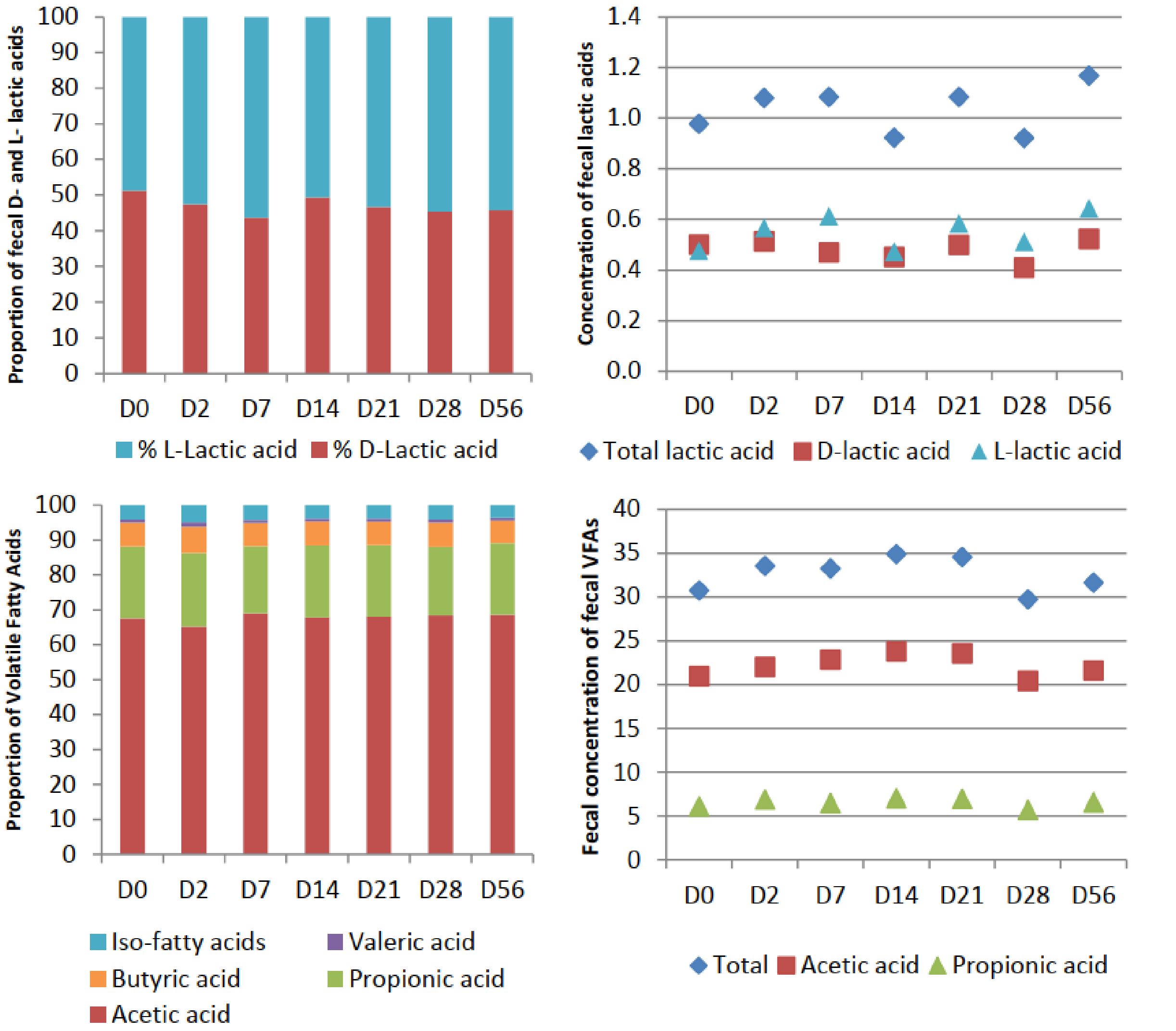

(A)

(B)

Figure 1. Days-related modifications of the proportions (%) and concentrations (mmol/L) of(A) 16lactic acids and (B) volatile fatty acids.
